# Supplementary material for: Pulmonary Ultrasonography in Systemic Sclerosis-Induced Interstitial Lung Disease—A Systematic Review and Meta-Analysis
Source: Diagnostics (Basel). 2023 Apr 16;13(8):1429. doi: 10.3390/diagnostics13081429 (PMC10138038; doi:10.3390/diagnostics13081429)
Supplement: Supplementary file 1 [file diagnostics-13-01429-s001.zip › diagnostics-2308386-supplementary.pdf]

Table S1. Detailed scoring in Revised Tool for the Quality Assessment of Diagnostic Accuracy Studies evaluation tool.

Author and year	Risk of bias				Applicability concerns		
	**Patient selection**	**Index test**	**Reference standard**	**Flow and timing**	**Patient selection**	**Index test**	**Reference standard**
Fairchild et al., 2021 [16]	Low	Low	Low	Low	Low	Low	Low
Gargani et al., 2020 [17]	Low	Low	Low	Unclear	Low	Low	Low
Hassan et al, 2019 [18]	Low	Low	Low	Low	Low	Low	Low
Tardella et al., 2018 [19]	Low	Low	Low	Low	Low	Low	Low
Çakır et al., 2016 [20]	Low	Low	High	Low	Low	Low	Low
Sperandeo et al., 2015 [21]	Low	Low	Low	Low	Low	Low	Low
Mohammadi et al., 2014 [22]	Low	Low	Low	Unclear	Low	Low	Low
Moazedi-Fuerst et al., 2014 [23]	Low	Low	Low	Unclear	Low	Low	Low
Barskova et al., 2013 [24]	Low	Low	Low	Low	Low	Low	Low
